# Psychological and Contextual Factors of AI Acceptance Among Informal Caregivers of People Living With Dementia: Cross-Sectional Pilot Study

**DOI:** 10.2196/104072

**Published:** 2026-09-30

**Authors:** Louis Fisher, Minh-Nguyet Hoang, Logan DuBose, Qiping Fan

**Affiliations:** 1 College of Behavioral, Social, and Health Sciences, Clemson University, 330 Hendrix, Clemson, SC, 29634, United States, 1 984-245-5244; 2 Naresh K. Vashisht College of Medicine, Texas A&M University, College Station, TX, United States; 3 Olera, Inc, Austin, TX, United States

**Keywords:** dementia, Alzheimer disease, family caregivers, artificial intelligence, psychological flexibility, artificial intelligence acceptance, attitude

## Abstract

**Background:**

Informal caregivers make up a large share of the care provided to people living with dementia, and experience an elevated risk for anxiety, depression, and caregiving burden. Technology-based interventions, such as mobile apps, aim to support informal caregivers by providing information, training, and mental or social support. Considering the diverse nature of the caregiver experience, AI is being incorporated into such interventions to create more tailored and specific support; however, AI hesitancy may serve as a significant barrier to usage.

**Objective:**

The study aimed to preliminarily assess the levels of AI acceptance in adult informal caregivers of people living with dementia, and generate hypotheses regarding the psychological and contextual factors of AI acceptance.

**Methods:**

Adult, unpaid caregivers were recruited through social media and community partners. A cross-sectional, web-based survey was administered through Qualtrics (Qualtrics, LLC) to evaluate AI acceptance and psychological flexibility (PF). AI acceptance was measured using the Attitude Towards Artificial Intelligence (ATTARI-12) scale, while PF was measured using the Personalized Psychological Flexibility Index (PPFI). Social needs were assessed by the ’Accountable Health Communities Health Related Social Needs (AHC-HRSN) tool (Centers for Medicare and Medicaid Services). Descriptive, correlational, and regression analyses were performed to examine the associations between these factors and AI acceptance.

**Results:**

Overall, 31 informal caregivers of people living with dementia completed the survey. With an average age of 60 (SD 10.6) years old, the majority of caregivers were women (29/31, 94%), Caucasian (25/31, 81%), highly educated (24/31, 77% completed some form of higher education), and currently serving in a caregiver role (21/31, 68%). Mean PPFI and ATTARI-12 scores were moderate (66.4, SD 10.3, and 3.01, SD 0.55, respectively), demonstrating a neutral attitude regarding AI use. In bivariate analyses, ATTARI-12 differed by the caregiver-perceived illness severity (*P*<.001). Linear regression suggested trends that the PPFI acceptance subscale (β=.05, 95% CI 0.002‐0.097; *P*=.04) was associated with ATTARI-12 scores.

**Conclusions:**

This study suggests that caregivers’ acceptance of such technology may vary according to their care recipient’s disease severity and the acceptance subscale of the PPFI. Additionally, the study provides justification for using the ATTARI-12 and PPFI scales to assess AI acceptance and PF in informal caregivers of people living with dementia, respectively. Future research should explore these factors across a larger and more diversified cohort to further generalize and confirm our findings.

## Introduction

### AI

AI has applications in many areas, especially in the medical and public health fields [1]. Despite its potential benefits, hesitancy to use AI has emerged among the general population, stemming from a complex array of anxieties arising from perceived risks, exposure to AI, and inherent anxiety about the uncertainty of AI [2]. Various demographic factors, such as age, gender, and education level, have been implicated as determinants of AI acceptance; however, varying results in the literature have been noted [3]. Psychological factors such as resilience and resistance to change have been demonstrated to produce negative and positive correlations with AI anxiety, respectively [4]. AI literacy, the understanding of AI functionality and utility, also correlates with greater AI acceptance [5], aligning with studies regarding self-rated AI knowledge [3]. Regarding health care, patients demonstrated hesitancy to use AI chatbots due to concern of information quality and trustworthiness, especially for more complex health issues and AI-supported decision-making [6,7]. AI-supported apps can become more acceptable if they demonstrate greater perceived usefulness, transparency, and ease of use [5,8]. Bolstering AI adoption may become increasingly important to lower usage barriers as the AI landscape continues to develop and expand. However, without understanding the underlying sources of AI acceptance within specific use cases or user populations, developers may lack the insights needed to design products that are acceptable and used.

### Dementia Caregiving Background

Alzheimer disease and related dementias (ADRD) prevalence is projected to nearly double by 2060, from 7.2 million to 13.8 million Americans aged 60 and older [9-12]. Family or informal caregivers constitute the majority of care provided for people living with dementia [12]; however, informal caregiving is associated with high levels of stress, burden, depression, and anxiety [13,14]. Psychological distress, further compounded by its associated adverse physical health effects [15], poses a significant threat to informal caregivers. Caregiver burden is shown to originate from various stressors, such as caregiver ideology and financial strain; however, the most notable among them is a lack of support and information [16-18]. Additionally, caregivers’ burdens differ depending on demographics, such as ethnicity, gender, and sexuality, each with unique barriers and challenges when caring for people living with dementia [12,19].

Support for informal caregivers exists; however, these support services are either not used due to unaccounted-for barriers or are not sufficiently meeting caregivers’ needs. Many identified caregiver interventions, ranging from in-person social programs to technological web-based or mobile educational apps, had similar limitations relating to an inability to provide caregivers with individualized, relevant, and quality support [20-22]. As such, interventions tailored to individual caregiver needs show greater effectiveness in reducing burden [23].

AI integration has various benefits for technology-based caregiver interventions; semantic refinement, real-time analysis, and personalized, tailored information may help informal caregivers better understand and apply information during decision-making [24]. AI has the potential to improve upon previous technology-based support interventions by using these advantages; however, it should be noted that lack of user-centric design and testing may impede efficacy in diverse caregiver contexts [25]. While some recent interventions have begun testing across end users during app development to mitigate such limitations [26], further research is needed on factors influencing usability and uptake of AI-integrated interventions for informal caregivers. Because acceptance of AI is a precondition for use, understanding its determinants is essential if AI-integrated tools are to reduce caregiver burden.

### Psychological Flexibility

Psychological flexibility (PF) can be defined as the ability and willingness of a person to change their thinking, behaviors, and actions in response to adversity [27]. PF has strong implications for public health, considering its trainable, dose-dependent protective effects against depression, anxiety, and stress [28]. As such, it is the primary theoretical model behind Acceptance and Commitment Therapy (ACT), which is shown to significantly reduce depressive symptoms in its participants [29]. PF relates directly to caregiver burden and distress, making it a useful characteristic to measure in the population. In informal caregivers of people living with dementia, PF was associated with decreased burden and better psychological outcomes [30]. This aligns with other studies investigating the role of PF in caregivers for various other chronic health conditions [31-33]. Additionally, PF has been shown to be an important factor in psychological resilience [34] and related to adaptability [35]. Furthermore, higher levels of psychological resilience have been correlated with greater ability to adapt [36]. Considering the theoretical framework of interconnections between PF, resilience, and adaptability, along with the aforementioned negative correlation between resilience and AI anxiety, PF represents a potential determinant of AI acceptance.

Considering its benefit to both psychological well-being in caregivers and potential relationship to AI acceptance of caregiver interventions, more research needs to be done on measuring levels of PF and its correlation to AI acceptance in informal caregivers. Accordingly, this study seeks to examine whether PF is associated with AI acceptance among informal caregivers of people living with dementia. Considering that PF can be increased through interventions such as ACT [29], if positive correlations between AI acceptance and PF are established, it could potentially justify ACT-based interventions as a means to bolster the adoption of AI-integrated support programs in caregivers. Lowering the barriers to usage of AI-integrated interventions could offer additional options for support for caregiver populations. We hypothesize that increased PF will be positively correlated with AI acceptance. Additionally, we hypothesize that sociodemographic factors, such as caregiver social need and perceived burden, will positively influence AI acceptance.

### Study Objectives

Our major aim is to inform future AI-integrated support interventions about factors affecting AI acceptance. This study will preliminarily assess the level of AI acceptance and PF in adult informal caregivers of people living with dementia. PF and other contextual factors will be investigated as potential determinants of AI acceptance to generate hypotheses for future investigations.

## Methods

### Study Design

This was a cross-sectional, web-based survey study. Reporting follows the Checklist for Reporting Results of Internet E-Surveys (CHERRIES) checklist for internet-based surveys and (Strengthening the Reporting of Observational Studies in Epidemiology) STROBE guidance for cross-sectional studies [37,38]. The completed checklists can be found in Checklists 1 and 2, respectively. Participant responses to a survey administered through Qualtrics (Qualtrics LLC) were screened for eligibility, assessed attitudes towards AI acceptance, and evaluated various factors that may influence their perception. The survey was developed using Qualtrics, LLC and reviewed to ensure functionality, clarity, and readability. The survey was open from mid-March to mid-April 2025, delivered across 11 pages. Survey items were kept to a minimum to avoid overwhelming participants, though pages with matrix tables had higher item numbers, not exceeding 20 items per page. Adaptive screening logic excluded respondents who did not meet eligibility criteria before survey items were displayed. Participants could review and change answers using back-navigation. Submissions were screened for duplicates by matching email addresses; any duplicate submissions were voided.

### Recruitment

Participants were recruited using electronic flyers (Multimedia Appendix 1) distributed through various social media outlets, nursing home online channels, and relevant online communities. Additionally, flyers were sent out in email lists, compiled through collaborative efforts with networks, such as Clemson Institute of Engaged Aging, the South Carolina Aging Research Network, Alzheimer’s Association South Carolina Chapter, and previous participants of studies with Olera, Inc. and Texas A&M University College of Public Health [16,26]. Interested individuals were encouraged to scan a QR code (available in the recruitment flyer and emails) or click a link (available in recruitment emails) to access the Qualtrics survey.

Following previous research methodology, inclusion criteria ensured only adult caregivers with a significant responsibility to the individual for whom they provide care (care recipient) were selected [30]. The inclusion criteria for caregivers in this study were that (1) they were at least 18 years old; (2) providing (or had provided) unpaid care for people living with dementia outside of a professional facility; and (3) demonstrated at least 2 of the following criteria: (a) has (or had) the most frequent contact with the person living with dementia, (b) assists (or assisted) in tasks of daily living, (c) helps (or helped) manage medical, legal, and/or financial decisions of the patient.

The survey contained questions that automatically screened research potential participants for eligibility. Responses were reviewed to ensure no duplicates were submitted, including any submitted after prior rejection by automated screening. If a duplicate was detected, by way of matching email addresses, both responses were voided from the final analysis. Responses were reviewed to ensure completeness, with incomplete surveys being excluded from analysis. Additionally, Qualtrics automatically flagged surveys if completed in less than 2 standard deviations below the mean completion time and were excluded from analysis due to the potential of low data quality.

### Assessments and Measurements

AI acceptance was measured using the Attitude Towards Artificial Intelligence (ATTARI-12) scale, a validated 12-item questionnaire with a high level of internal consistency across samples with differing ages and backgrounds (Cronbach α=0.93) [39]. The survey responses were based on a 5-point Likert scale, ranging from strongly disagree (1) to strongly agree (5). Items indicating a negative perception of AI were reverse-coded so that higher scores predict greater AI acceptance, with 3.0 indicating a neutral mid-point [39]. A mean value across all 12 items produced a single score on a scale of AI aversion (1) to AI enthusiasm (5). Additionally, the scale did not emphasize any one particular use case of AI, and is, therefore, applicable across various research disciplines.

The main psychological factor tested in this study, PF, was measured using the Personalized Psychological Flexibility Index (PPFI), a more recent tool that was developed to improve upon previous measures of PF, such as the widely used Acceptance and Action Questionnaire-II (AAQ-II) [40], and is a promising measure of the construct [41,42]. The PPFI is a psychometrically validated 15-item questionnaire with good internal consistency across various adult US nonclinical samples (Cronbach α=0.82) [41,42]. The PPFI used a 7-point Likert scale to measure 3 separate subscales: acceptance (PPFIac), avoidance (PPFIav), and harnessing (PPFIh). Each subscale has 5 associated items scored from strongly disagree (1) to strongly agree (7), while the avoidance subscale is reverse-coded. Scores are summed together with higher values indicating greater acceptance, harnessing, and less avoidance, respectively. An overall score from 15 to 105 can be calculated across the subscales, with higher scores indicating greater PF.

Social need factors across 5 domains (housing and food insecurity, transportation and utility needs, and interpersonal safety) were screened for using the Accountable Health Communities Health-related Social Needs (AHC-HRSN) tool developed by the Centers for Medicare & Medicaid Services [43]. This is a 10-item screening tool that was developed in order to identify social need factors that negatively impact health care usage [43]. Housing needs are assessed on a yes or no binary and are indicated if the respondent selects any option other than “I have housing” and “none of the above” for items 1 and 2, respectively. Food insecurity is similarly indicated by selecting an option other than “never true” for either item. For both transportation and utility needs, selection of an option other than “no” would indicate an unmet need. Finally, 4 items assess interpersonal safety on a 5-point scale from never (1) to frequently (5), with total scores ranging from 4 to 20. Summed interpersonal safety responses greater than 10 indicate a threat of interpersonal violence.

Demographic characteristics were collected, including age, gender identity, race or ethnicity, marital status, employment status, self-perceived financial status, and education level. Caregiver-specific characteristics were also collected, including caregiver relationship to people living with dementia, caregiving duration, self-perceived illness severity, other work or family obligations, and weekly caregiving hours. These items were adapted from prior methodology in the literature [30].

### Ethical Considerations

This study was reviewed and approved by the Clemson University Institutional Review Board (IRB No. 2024‐0562). Because informal caregivers of persons living with dementia may experience elevated stress and burden, protections appropriate to a potentially vulnerable population were applied. Eligible individuals reviewed an electronic consent form outlining the study’s purpose, procedures, risks, benefits, confidentiality, voluntariness, and contact information before any survey items were presented. Affirmative consent was required to proceed. Responses were collected through Qualtrics and subsequently deidentified. Email addresses used for duplicate detection and incentive drawing were stored separately from survey data. As compensation, participants who completed the survey were entered into a drawing for one of 20 US $25 Visa gift cards. No personally identifying information is reported.

### Data Analysis

Analyses were conducted in Microsoft Excel and R 4.4.1 (R Foundation). Current and former caregivers were retained in the primary analyses. Considering the diversity in settings and roles caregiving encompasses, caregiver identity is dynamic and extends beyond active care [44]. Additionally, AI acceptance is attitudinal in nature, not behavioral; therefore, it is less contingent on active caregiver status. Consistent with this rationale, preliminary and sensitivity analyses were conducted to ensure minimal meaningful difference between current and former caregivers. Participant characteristics were reported as counts and percentages for categorical variables and as means and SDs for continuous variables. Bivariate comparisons used independent-samples *t*-tests for dichotomous grouping variables and one-way ANOVA for comparisons involving 3 or more groups, with normality assessed by inspection of residual distributions. Benjamini-Hochberg correction was applied to control the false discovery rate across predictors. Associations between PPFI subscales and ATTARI-12 were examined using univariable linear regression. Post hoc regression analyses were conducted for perceived illness severity and financial status given their hypothesis generating interest as potential predictors with ATTARI-12 seen in the ANOVA results. Both predictors were ordinal with a small number of categories, so we used dummy-coded regression to characterize the direction and magnitude of pairwise differences relative to the respective reference groups.

Because this was an exploratory pilot study intended to describe AI acceptance and generate preliminary estimates of associations in an understudied population; recruitment was based on feasibility rather than on a confirmatory a priori power calculation. The final sample size of 31 participants is not sufficient to reliably detect small-to-moderate associations or subgroup differences. For context, a two-sample *t*-test designed to detect a moderate effect would require more participants than were available in the present study, and one-way ANOVA models with 3 or more groups would require larger samples. Therefore, all inferential analyses are interpreted as exploratory and hypothesis-generating, with emphasis placed on effect-size estimates, CIs, and the direction of observed associations rather than statistical significance. Furthermore, while effect size estimates may be imprecise, the major aim of this study is to inform hypothesis generation for large sample-size studies.

## Results

### Participants’ Characteristics

As seen in Figure 1, out of 876 research leads that were emailed coupled with other advertisement outreach for study recruitment, only 54 individuals interacted with the survey (54/876, 6.16%). Of them, 20.4% (11/54) did not meet the eligibility criteria and were excluded. A total of 31 informal caregivers completed all survey items, with an average duration of 18 (SD 7.3) minutes. Of them, the majority were current caregivers (Table 1). The participants were predominantly women and White or Caucasian. The cohort was also highly educated, with all obtaining a high school diploma or equivalent, and the majority of participants completing at least some form of postsecondary education (24/31, 77%). The caregivers were experienced and highly burdened, reporting a range of 2‐23 years of caregiving and high average hours per week of caregiving, though this number was highly variable. Additionally, the majority of caregivers indicated that their care recipient’s dementia status was “moderately severe” to “very severe” (22/31, 71%).

**Figure 1.**
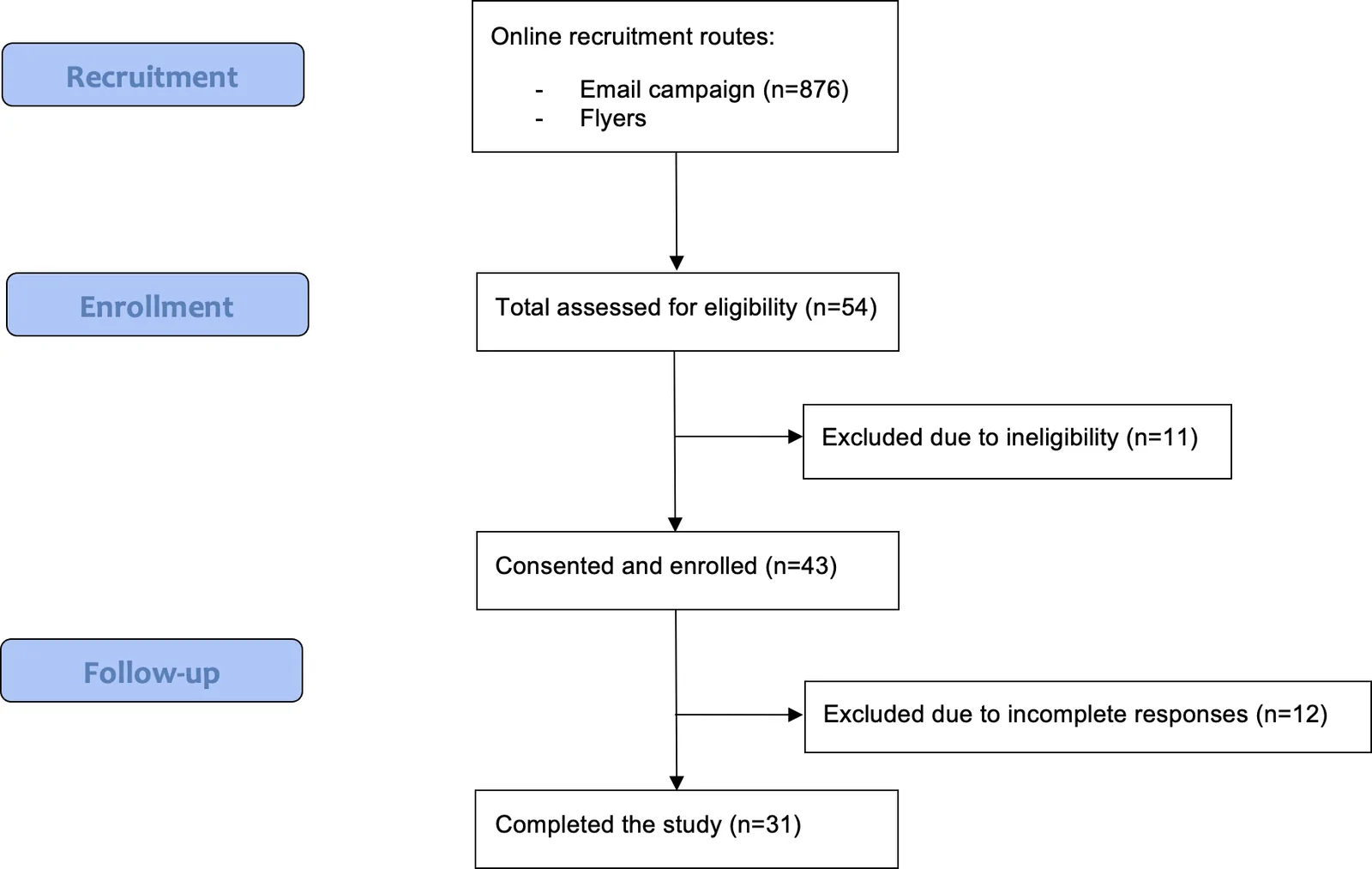
Participant flow diagram of recruitment, enrollment, and completion of the study.

**Table 1.** Caregiver demographics and characteristics among caregiving status.

Characteristics	All caregivers^a^	Current caregivers^b^	Past caregivers^c^
Age (years), mean (SD)	60 (10.6)	61 (9)	57 (13.4)
Years caregiving, mean (SD)	6.6 (5.18)	6.9 (5.24)	5.9 (5.26)
Hours caregiving per week, mean (SD)	74.2 (53.34)	69.6 (56.01)	83.8 (48.6)
Gender, n (%)			
Women	29 (94)	20 (95)	9 (90)
Men	2 (6)	1 (5)	1 (10)
Other (Nonbinary, Other)	0 (0)	0 (0)	0 (0)
Race, n (%)			
White or Caucasian	25 (81)	16 (76)	9 (90)
Black or African American	6 (19)	5 (24)	1 (10)
Education, n (%)			
High school degree or equivalent	1 (3)	0 (0)	1 (10)
Some college	6 (19)	3 (14)	3 (30)
Associates degree	4 (13)	3 (14)	1 (10)
Bachelor’s degree	13 (42)	11 (52)	2 (20)
Graduate degree	7 (23)	4 (19)	3 (30)
Marital status, n (%)			
Married	16 (52)	11 (52)	5 (50)
Divorced	6 (20)	5 (24)	1 (10)
Widowed	1 (3)	0 (0)	1 (10)
Never married	8 (26)	5 (24)	3 (30)
Relationship to care recipient, n (%)			
Spouse	7 (23)	6 (29)	1 (10)
Parent or legal guardian	9 (29)	5 (24)	4 (40)
Child	9 (29)	7 (33)	2 (20)
Other family	6 (19)	3 (14)	3 (30)
Perceived illness severity, n (%)			
Very severe	3 (10)	1 (5)	2 (20)
Severe	6 (19)	2 (10)	4 (40)
Moderately severe	13 (42)	10 (48)	3 (30)
Moderately mild	8 (26)	7 (33)	1 (10)
Mild	1 (3)	1 (5)	0 (0)
Very mild	0 (0)	0 (0)	0 (0)

a n=31.

b n=21.

c n-10.

The caregivers scored an average of 66.4 (SD 10.3) on the PPFI and a 3.01 (SD 0.55) on the ATTARI-12 (Multimedia Appendix 2). Total averages of the PPFI Avoidance and Acceptance subscales were similar (23.9 and 25.9, respectively), while the harnessing subscale was lower (16.7; Table 2). In Table 2, differences between past and current caregivers in the main theoretical constructs, total PF and AI acceptance, were not statistically significant. Notably, however, past caregivers demonstrated significantly higher PPFI acceptance subscale scores compared to current caregivers (Cohen *d*=1.078, *P*=.009), indicating a meaningful difference, which is discussed in conjunction with the sensitivity analysis below. Additionally, between-group comparisons for PF of the full sample revealed significant differences according to financial status and food insecurity designation (Multimedia Appendix 3).

**Table 2.** *T*-test comparisons of AI acceptance and psychological flexibility (PF) among current and past caregivers.

	Current caregivers	Past caregivers	Summary statistics			
Variables	Mean (SD)	Mean (SD)	Difference	*t* test (*df*)	Cohen *d*	*P* values
ATTARI-12^a^	2.93 (0.52)	3.19 (0.58)	− 0.26 (−0.722 to 0.196)	−1.21 (16.2)	0.453	.242
PPFI^b^ Total	64.6 (10.4)	70.4 (9.1)	− 5.8 (−13.4 to 1.88)	−1.57 (20.2)	0.587	.131
PPFI^c^ Ac	24.7 (3.9)	28.5 (3.2)	− 3.8 (−6.60 to −1.07)	−2.88 (21.1)	1.078	.009
PPFI^d^ Av	22.6 (7.0)	26.7 (7.5)	− 4.1 (−10.10 to 1.84)	−1.46 (16.7)	0.546	.163
PPFI^e^ H	17.4 (5.6)	15.2 (3.7)	2.2 (−1.36 to 5.72)	1.27 (24.7)	0.475	.216

a ATTARI-12: Attitudes toward Artificial Intelligence 12-item scale.

b PPFI: Personalized Psychological Flexibility Index

c PPFI Ac: Personalized Psychological Flexibility Index acceptance subscale.

d PPVI Av: Personalized Psychological Flexibility Index avoidance subscale.

e PPFI H: Personalized Psychological Flexibility Index harnessing subscale.

### AI Acceptance Covariates

AI acceptance (ATTARI-12) scores were taken as an average across all scale items, with higher scores corresponding to greater AI acceptance (Table 3). Between-group comparisons were investigated using both *t*-tests and ANOVAs, depending on characteristic stratification. AI acceptance was found to be different depending on the participants’ self-rated perception of the severity of their care recipient’s dementia (*F*_2,28_=11.36, ƞ²=0.448, *P*<.001). This relationship remained significant after Benjamini-Hochberg correction (*p*-adjusted=0.013). Those who rated the illness severity as the most severe (“severe” or “very severe”) demonstrated the highest level of AI acceptance. The mildest group (“mild” or “moderately mild”) had the second highest ATTARI-12 score, while the moderate group (“moderately severe”) had the lowest. Financial status was not significantly associated with AI acceptance; however, the lowest income group (“lower” or “upper lower”) had the lowest mean ATTARI-12 scores (2.63), compared to the higher income groups with average scores of 3.09 and 3.19 (*F*_2,28_=3.22, ƞ²=0.187, *P*=.055). While not significant, compared to the other results within the sample, the relatively large effect size and borderline *P* value demonstrated hypothesis-generating results worthy of follow-up. No significant differences in AI acceptance were observed across age, education, or social need status (Table 3). Full summary statistics for all between-group comparisons are reported by test type and variable (Multimedia Appendix 4).

**Table 3.** Mean ATTARI-12^a^ scores across sociodemographic, caregiver, and social-need subgroups with intergroup comparisons reported as *t*-test or ANOVA *P* values.

Characteristic	ATTARI-12^b^ Mean (SD)	Test Statistics *t or F* test (*df)*	Group Comparisons *P* (Cohen *d* or ƞ²)
Age		−0.65 (17.86)	0.522 (0.237)
Less than 60 years (n=13)	3.10 (0.71)		
60+ years (n=18)	2.95 (0.41)		
Education		1.51 (2,28)	0.239 (0.097)
Less than a bachelor’s degree (n=11)	3.02 (0.52)		
Bachelor’s degree (n=13)	2.86 (0.57)		
Graduate degree (n=7)	3.30 (0.51)		
Perceived illness severity		11.36 (2,28)	<0.001^c^ (0.448)
Severe to very severe (n=9)	3.51 (0.52)		
Moderately severe (n=13)	2.64 (0.34)		
Mild to moderately mild (n=9)	3.06 (0.42)		
Marriage status		1.01 (2,28)	0.376 (0.068)
Married (n=16)	2.92 (0.54)		
Separated (n=7)	2.95 (0.44)		
Never married (n=8)	3.25 (0.63)		
Employment status		0.93 (2,28)	0.407 (0.062)
Part-time (n=10)	2.91 (0.82)		
Full-time (n=7)	2.87 (0.31)		
Not employed (n=14)	3.16 (0.37)		
Financial status		3.22 (2,28)	0.055 (0.187)
Lower to upper lower (n=8)	2.63 (0.53)		
Lower middle (n=13)	3.19 (0.47)		
Upper middle to upper (n=10)	3.09 (0.55)		
Housing needs		−0.55 (14.05)	0.590 (0.218)
Yes (n=9)	3.10 (0.58)		
No (n=22)	2.98 (0.54)		
Food insecurity		−0.45 (9.90)	0.665 (0.193)
Yes (n=7)	3.10 (0.55)		
No (n=24)	2.99 (0.56)		
Transport or utility needs		−0.46 (7.25)	0.659 (0.209)
Yes (n=6)	3.11 (0.59)		
No (n=25)	2.99 (0.55)		

a ATTARI-12: Attitude Towards Artificial Intelligence.

b ATTARI-12: Attitudes toward Artificial Intelligence 12-item scale.

c Significant (*P*<.05) after Benjamini-Hochberg correction for multiple comparisons.

### Regression Analysis of AI Acceptance and PF

Simple linear regression was used to determine if any of our recorded variables could predict levels of AI acceptance (Table 4). Given the limited sample size, only univariable models were performed with no adjustment for potential confounders. Regarding PF, the analysis revealed a significant positive relationship between the acceptance subscale of the PPFI (PPFIac) and ATTARI-12 scores. PPFIac explained 13.5% of the variation in ATTARI-12 scores (*F*_1,29_=4.54; *P*=.04). Additionally, while not statistically significant, we observed a similar direction and magnitude of association in the harnessing subscale of the PPFI (PPFIh) with AI acceptance (*F*_1, 29_=3.32; *P*=.08).

Given the ordinal nature of the perceived illness severity of the care recipient and the significant ANOVA result with AI acceptance scores, supplementary linear regression was applied to further examine the relationship. Using the mild group as the reference group, the directionality and linearity of the relationship between perceived illness severity and ATTARI-12 scores were tested. Despite significant differences in ATTARI-12 scores between the groups, the relationship was not linear. As caregiver perception increased to moderate severity, their ATTARI-12 score decreased by an average of 0.415 (*P*=.03); however, as caregiver perception progressed to the severe group, scores actually increased by an average of 0.454 (*P*=.03). This relationship explained nearly half of the variance in ATTARI-12 scores (*R*^2^=0.4479, *F*_2,28_=11.36, *P*<.001). A similar analysis was conducted with financial status, though the findings were not statistically significant. Using the highest perceived income bracket as the reference group, as perceived income decreased to the middle bracket, ATTARI-12 scores decreased by 0.467 (*P*=.07); however, at the lowest income level, scores were higher by 0.101 (*P*=.64).

Sensitivity analyses through the restriction of all primary analyses to current caregivers (n=21) yielded directionally consistent findings across all models (Multimedia Appendix 5). ATTARI-12 associations with illness severity were maintained and strengthened in the restricted sample with the moderately severe group (β*=*−.404, *P*=.04) and severe group (β=.701, *P*=.01), demonstrating comparable effects to the full sample. The PPFIac association with ATTARI-12 yielded identical effect sizes (β=.050) but lost statistical significance (*P*=.08), consistent with the reduced statistical power expected of the smaller sample size. Financial status associations were the most affected in the restricted sample, warranting greater caution in interpretation.

**Table 4.** Univariable linear regression models predicting ATTARI-12^a^ scores from psychological flexibility, perceived illness-severity, and financial status.

Univariate models	*R* ^2^	*F* test (*df*)	β	SE	95% CI	*t* test (*df*)	*P* values
PF^b^							
PPFI^c^	0.080	2.51 (1,29)	.015	0.010	(−0.004, 0.035)	1.59 (29)	.12
PPFI^d^ ac	0.135	4.54 (1,29)	.050	0.023	(0.002, 0.097)	2.13 (29)	.042
PPFI^e^av	0.001	0.03 (1,29)	−.002	0.014	(−0.031, 0.026)	−0.17 (29)	.87
PPFI h^f^	0.103	3.32 (1,29)	.034	0.019	(−0.004, 0.073)	1.82 (29)	.08
Illness severity^g^							
Moderately severe	—^h^	—	−.415	0.183	(−0.789 to−0.040)	−2.27 (29)	.03
Severe to very severe	—	—	.454	0.199	(0.047, 0.861)	2.28 (29)	.03
Financial status^i^							
Lower to upper lower	—	—	−.467	0.243	(−0.963, 0.030)	−1.93 (29)	.06
Lower middle	—	—	.101	0.215	(−0.340, 0.541)	0.47 (29)	.64

a ATTARI-12: Attitudes toward Artificial Intelligence 12-item scale.

b PF: psychological flexibility.

c PPFI: Personalized Psychological Flexibility Index.

d PPFI ac: Personalized Psychological Flexibility Index acceptance subscale.

e PPVI av: Personalized Psychological Flexibility Index avoidance subscale.

f PPFI h: Personalized Psychological Flexibility Index harnessing subscale.

g Reference: mild to moderately mild (overall: *R*2=0.448, *F*_2,28_=11.36, *P*<.001).

h Not applicable.

i Reference: upper middle to upper (overall: *R*2=0.187, *F*_2,28_=3.22, *P*=.055).

## Discussion

### Principal Findings

By applying the PPFI and ATTARI-12 in this population, this study offers preliminary insight into social and psychological factors that may shape informal dementia caregivers’ uptake of AI-integrated tools. The overall moderate ATTARI-12 score demonstrated a neutral attitude regarding AI use. Additionally, various factors have been identified as potential determinants of caregiver AI acceptance, including self-perceived illness severity of their care recipient, self-perceived financial status, and PF. The estimated effect sizes of the described factors are likely imprecise due to a limited sample size; however, they generate hypotheses for future work.

AI acceptance varied most with caregiver-rated dementia severity and remained statistically significant after correction. The relationship between AI acceptance and perceived illness severity was not linear. Participants who rated their care recipients as having the highest level of severity demonstrated the highest level of AI acceptance. It is important to note that participants may have consciously or subconsciously conflated AI acceptance with acceptance of AI-integrated caregiver support interventions. While the ATTARI-12 presented questions in a generalized manner, the context of the study was regarding caregiving and AI integration of caregiving services. Therefore, participants who reported the highest severity level of dementia in their persons living with dementia may have indicated greater need for caregiving services and, thus, greater levels of AI acceptance or acceptance of AI-integrated interventions. Furthermore, the group with moderate levels of perceived severity demonstrated the lowest level of AI acceptance, while the mild severity group demonstrated intermediate AI acceptance.

One tentative interpretation is that the mild severity group has less need for AI caregiving services than the high severity group, though they perceive less risk in using them due to managing milder symptoms. Conversely, the moderate severity group may perceive more risk in using AI caregiving services as they manage more severe symptoms with higher stakes, though they are not at the stage of the severe groups where they perceive enough need for support to overcome that perceived risk for AI. A caregiver’s perception of illness severity is variable across a complex range of factors [45], and, therefore, may differ from clinical criteria of dementia severity. However, as AI acceptance is attitudinal in nature, perception may be more influential as a determinant than clinical assessments, though future study is needed to investigate those relationships. It is also important to note that the skewed distribution and small cell sizes across caregiver-perceived severity levels (n=3, 6, 13, 8, 1, 0) necessitated collapsing the original 6 levels into 3 broader groups (n=9, 13, 9). Considering the still small cell sizes and potential obscuring of differences between adjacent severity levels, these findings should be interpreted with caution.

### Comparison With Prior Work

While relatively new, several prior studies have demonstrated that the ATTARI-12 scale has good validity and reliability in assessing AI attitudes in the general population internationally [46,47]. Our sample demonstrated neutral acceptance toward AI (mean 3.01, SD 0.55). Stein et al [39] reported a higher average ATTARI-12 score of 3.66 (SD 0.83), though it is important to note that the sample was younger and not representative of US dementia caregivers. The overall average of PF scores among all caregivers was moderate (66.4, SD 10.3), above the scale’s midpoint at approximately 56% of the scale’s maximum. To our knowledge, previous assessments of PF in dementia caregivers have primarily used the AAQ-II, which differs in both range and directionality compared to the PPFI. The AAQ-II is scored from 7‐49, with higher scores demonstrating lesser PF, while the PPFI is scored from 15‐105, with higher scores indicating greater PF [40,41]. In informal caregiver samples, Han et al and Tan et al [33,48] reported directionally consistent mean AAQ-II scores both below the scale’s midpoint (22.45 and 20.88, respectively), indicative of moderate PF that aligned with our sample. Given the limited use of the PPFI in this population, comparisons with prior AAQ-II-based studies provide indirect contextual support, as both indicate moderate levels of PF-related functioning.

Perceived financial status represented hypothesis-generating findings worthy of investigation in further, more adequately powered studies. While not significant at this sample size, participants who reported lower financial status demonstrated the lowest level of AI acceptance. Those who reported moderate or high financial status had similar levels of AI acceptance, suggesting a plateau in acceptance at higher levels of perceived financial status. It is important to note that financial status was self-perceived by caregivers, and, therefore, may not align with standardized income thresholds, introducing additional variability. There is currently little literature regarding the effect of socioeconomic status on AI acceptance specifically; however, the lower levels of AI acceptance observed in individuals with lower perceived financial status align with prior literature demonstrating disparities in access to digital health technologies among lower socioeconomic groups [49].

We found no significant associations between AI acceptance and age, education level, or social need factors. Contrary to our findings, previous literature reported that younger age and higher levels of education are associated with more positive AI attitudes [50]. Social need factors, such as housing and food insecurity, transportation and utility needs, or interpersonal safety, were assessed by the AHC-HRSN screening tool; however, no significant relationships with AI acceptance were demonstrated. This contrasted with previous work demonstrating a moderate positive effect of socioeconomic status on perceptions of AI [51].

### Limitations and Implications

This study had several limitations. First, the smaller sample size limits generalizability and precludes causal inference. The lower statistical power afforded by our sample size means our findings should be interpreted as exploratory in nature to give stronger justification for further investigation. Considering the smaller sample size, controlling for confounders through multiple regression was methodologically impractical; therefore, our findings do not reflect independent effects. Furthermore, regression coefficients should be interpreted as preliminary estimates due to the study’s model instability and the results’ wide CIs from our lower sample size. Additionally, inclusion of former caregivers complicates interpretation since attitudes toward AI and AI-integrated caregiving tools may differ between those actively engaged in caregiving and those who have concluded that role. While group comparisons revealed equivalence in primary outcomes and sensitivity analyses suggested directionally consistent findings in current caregivers only, differences in the acceptance subscale of the PPFI suggest significant discrepancy between the populations.

Second, participants were entered into a drawing to receive US $25 Visa Gift cards. While not having guaranteed participant incentives may have reduced the likelihood of incentive-related response bias, it was also a probable contributor to difficulties recruiting participants, resulting in our smaller sample size. Third, the cross-sectional design cannot establish temporal sequence between PF, social needs, and AI acceptance. Finally, participants were mainly recruited from listservs from previous studies conducted by study personnel [16,26] and through aging-focused community partners, so there may be limitations in participant selection. Specifically, recruiting from prior study participant pools may have yielded a sample that is more research-engaged, more digitally literate, or more familiar with existing caregiving support infrastructures than the general caregiver population, which may limit generalizability. Additionally, the demographics of the study cohort are predominantly women, White, and highly educated. While this demographic composition reflects the broader population of informal caregivers in the United States [12], our sample lacks the diversity necessary for a representative sample and may limit the generalizability of the results.

Despite these limitations, the key strength in this study lies in the novel integration of 2 important scales when considering caregiver populations. The ATTARI-12 and PPFI are newer scales, designed and verified to improve on older measures for AI acceptance and PF, respectively [39,41]. While PF has commonly been studied in caregiver communities due to its protective effects against stress and anxiety, AI acceptance may become increasingly important as AI is further implemented into caregiver interventions. Additionally, the understanding of factors impacting caregiver acceptance of AI-integrated tools can inform intervention designers about methods to increase usage. For example, our preliminary findings suggest that caregivers may be more or less likely to accept AI-integrated tools according to their care recipient’s dementia progression. As this relationship is not linear, AI-integrated intervention development may benefit from consideration of disease status in its design, presentation, and functionalities. Furthermore, preliminary evidence of relationships between PPFI subscales and AI acceptance may suggest inclusion of onboarding ACT-based modules within AI-integrated interventions to improve AI attitudes, while also targeting intervention outcomes by bolstering caregiver psychological well-being.

### Future Directions

Future studies should be conducted with a larger and more diverse cohort to further generalize and explore informal caregivers’ attitudes toward AI-assisted caregiving support and resources. Studies can further investigate these relationships with higher statistical power while also confirming additional factors previously explored in the literature, such as AI literacy and previous exposure to AI. Longitudinal designs examining whether PF precedes changes in AI acceptance over time and whether ACT-based modules within AI-integrated interventions improve acceptance would help confirm the hypothesized relationships identified in this pilot study across larger and more diverse caregiving populations. Future studies with sufficient sample sizes should also analyze current and former caregivers as independent groups to characterize differences between them regarding these outcomes. It is also important to note that one of our main findings, the relationship between perceived illness severity of the care recipient and AI acceptance, is based on the caregiver’s subjective rating. Future studies should include more objective measures of care recipient illness severity to further investigate this relationship.

### Conclusions

While the benefits of AI-integrated technology in supporting informal caregivers of people living with dementia are evident, our study provides preliminary evidence that caregivers’ acceptance of such technology may potentially be influenced by their care recipient’s disease severity and the acceptance subscale of the PPFI. Our preliminary findings inform the importance of addressing these factors in the development of AI-integrated caregiving support tools. Additionally, we used the novel ATTARI-12 and PPFI as our evaluative tools. Future research should explore these factors across a larger and more diversified cohort to further generalize our findings.

## Supplementary material

10.2196/104072 Multimedia Appendix 1 Flyer used for participant recruitment.

10.2196/104072 Multimedia Appendix 2 Average ATTARI-12 and PPFI scores among caregivers.

10.2196/104072 Multimedia Appendix 3 Between group comparisons of PPFI across various factors.

10.2196/104072 Multimedia Appendix 4 Summary statistics for ATTARI-12 and PPFI ANOVAs across various factors.

10.2196/104072 Multimedia Appendix 5 Sensitivity analysis for ATTARI-12 and PPFI across various factors.

10.2196/104072 Checklist 1 Completed CHERRIES guideline checklist for internet e-surveys.

10.2196/104072 Checklist 2 Completed STROBE guideline checklist for cross-sectional studies.

## Abbreviations

AAQ-II: Acceptance and Action Questionnaire-II
ACT: Acceptance and Commitment Therapy
ADRD: Alzheimer Disease and related dementias
AHC-HRSN: Accountable Health Communities Health Related Social Needs
ATTARI-12: Attitude Towards Artificial Intelligence
CHERRIES: Checklist for Reporting Results of Internet E-Surveys
PF: psychological flexibility
PPFI: Personalized Psychological Flexibility Index
PPFIac: Personalized Psychological Flexibility Index acceptance subscale
PPFIav: Personalized Psychological Flexibility Index avoidance subscale
PPFIh: Personalized Psychological Flexibility Index harnessing subscale
STROBE: Strengthening the Reporting of Observational Studies in Epidemiology

## References

[1] Younis HA, Eisa TAE, Nasser M (2024). A systematic review and meta-analysis of artificial intelligence tools in medicine and healthcare: applications, considerations, limitations, motivation and challenges. Diagnostics (Basel).

[2] Li J, Huang JS (2020). Dimensions of artificial intelligence anxiety based on the integrated fear acquisition theory. Technol Soc.

[3] Kaya F, Aydin F, Schepman A, Rodway P, Yetişensoy O, Demir Kaya M (2024). The roles of personality traits, AI anxiety, and demographic factors in attitudes toward artificial intelligence. Int J Hum-Comput Interact.

[4] Kenku A, Uzoigwe TL (2024). Determinants of artificial intelligence anxiety: Impact of some psychological and organisational characteristics among staff of Federal Polytechnic Nasarawa, Nigeria. Afr J Psychol Study Soc Issues.

[5] Schiavo G, Businaro S, Zancanaro M (2024). Comprehension, apprehension, and acceptance: Understanding the influence of literacy and anxiety on acceptance of artificial Intelligence. Technol Soc.

[6] Nadarzynski T, Miles O, Cowie A, Ridge D (2019). Acceptability of artificial intelligence (AI)-led chatbot services in healthcare: a mixed-methods study. Digit Health.

[7] Rojahn J, Palu A, Skiena S, Jones JJ (2023). American public opinion on artificial intelligence in healthcare. PLoS ONE.

[8] Ismatullaev UVU, Kim SH (2024). Review of the factors affecting acceptance of AI-infused systems. Hum Factors.

[9] Cao Q, Tan CC, Xu W (2020). The prevalence of dementia: a systematic review and meta-analysis. J Alzheimers Dis.

[10] Prince M, Guerchet M, Prina M (2013). The global impact of dementia 2013-2050.

[11] Ferri CP, Prince M, Brayne C (2005). Global prevalence of dementia: a Delphi consensus study. Lancet.

[12] Alzheimer’s Association (2025). 2025 Alzheimer’s disease facts and figures. Alzheimers Dement.

[13] Frias CE, Cabrera E, Zabalegui A (2020). Informal caregivers’ roles in dementia: the impact on their quality of life. Life (Basel).

[14] Collins RN, Kishita N (2020). Prevalence of depression and burden among informal care-givers of people with dementia: a meta-analysis. Ageing Soc.

[15] Pinquart M, Sorensen S (2007). Correlates of physical health of informal caregivers: a meta-analysis. J Gernotol B Psychol Sci Soc Sci.

[16] Fan Q, DuBose L, Ory MG (2023). Financial, legal, and functional challenges of providing care for people living with dementia and needs for a digital platform: interview study among family caregivers. JMIR Aging.

[17] Hellis E, Mukaetova-Ladinska EB (2023). Informal caregiving and Alzheimer’s disease: the psychological effect. Medicina (B Aires).

[18] Lindeza P, Rodrigues M, Costa J, Guerreiro M, Rosa MM (2020). Impact of dementia on informal care: a systematic review of family caregivers’ perceptions. BMJ Support Palliat Care.

[19] Morgan E, Dyar C, Feinstein BA, Rose K (2024). Sexual and gender minority differences in likelihood of being a caregiver and levels of caregiver strain in a sample of older adults. J Homosex.

[20] Wiegelmann H, Speller S, Verhaert LM, Schirra-Weirich L, Wolf-Ostermann K (2021). Psychosocial interventions to support the mental health of informal caregivers of persons living with dementia - a systematic literature review. BMC Geriatr.

[21] Yu Y, Xiao L, Ullah S (2023). The experiences of informal caregivers of people with dementia in web-based psychoeducation programs: systematic review and metasynthesis. JMIR Aging.

[22] Ye B, Chu CH, Bayat S, Babineau J, How TV, Mihailidis A (2023). Researched apps used in dementia care for people living with dementia and their informal caregivers: systematic review on app features, security, and usability. J Med Internet Res.

[23] Beinart N, Weinman J, Wade D, Brady R (2012). Caregiver burden and psychoeducational interventions in Alzheimer’s disease: a review. Dement Geriatr Cogn Dis Extra.

[24] Milella F, Russo DD, Bandini S (2023). AI-Powered solutions to support informal caregivers in their decision-making: a systematic review of the literature. OBM Geriatr.

[25] Wang L, Smriti D, Yuan H, Huh-Yoo J Artificial intelligence systems for supporting informal caregivers of people living with alzheimer’s disease or related dementias: a systematic review.

[26] Fan Q, Hoang MN, DuBose L (2024). The Olera.care digital caregiving assistance platform for dementia caregivers: preliminary evaluation study. JMIR Aging.

[27] Doorley JD, Goodman FR, Kelso KC, Kashdan TB (2020). Psychological flexibility: what we know, what we do not know, and what we think we know. Social Personality Psych.

[28] Gloster AT, Meyer AH, Lieb R (2017). Psychological flexibility as a malleable public health target: evidence from a representative sample. J Contextual Behav Sci.

[29] Bai Z, Luo S, Zhang L, Wu S, Chi I (2020). Acceptance and Commitment Therapy (ACT) to reduce depression: a systematic review and meta-analysis. J Affect Disord.

[30] Tan KP, Ang JK, Koh EBY, Pang NTP, Mat Saher Z (2023). Relationship of psychological flexibility and mindfulness to caregiver burden, and depressive and anxiety symptoms in caregivers of people with dementia. Int J Environ Res Public Health.

[31] Lappalainen P, Keinonen K, Pakkala I, Lappalainen R, Nikander R (2021). The role of thought suppression and psychological inflexibility in older family caregivers’ psychological symptoms and quality of life. J Contextual Behav Sci.

[32] Klietz M, Drexel SC, Schnur T (2020). Mindfulness and psychological flexibility are inversely associated with caregiver burden in Parkinson’s disease. Brain Sci.

[33] Jansen JE, Haahr UH, Lyse HG, Pedersen MB, Trauelsen AM, Simonsen E (2017). Psychological flexibility as a buffer against caregiver distress in families with psychosis. Front Psychol.

[34] McCracken LM, Badinlou F, Buhrman M, Brocki KC (2021). The role of psychological flexibility in the context of COVID-19: Associations with depression, anxiety, and insomnia. J Contextual Behav Sci.

[35] Waldeck D, Pancani L, Holliman A, Karekla M, Tyndall I (2021). Adaptability and psychological flexibility: overlapping constructs?. J Contextual Behav Sci.

[36] Demetriou L, Drakontaides M, Hadjicharalambous D (2020). Psychological resilience hope and adaptability as protective factors in times of crisis: a study in greek and cypriot society during the COVID-19 pandemic. SSRN.

[37] Eysenbach G (2004). Improving the quality of web surveys: the checklist for reporting results of internet E-surveys (CHERRIES). J Med Internet Res.

[38] Cuschieri S (2019). The STROBE guidelines. Saudi J Anaesth.

[39] Stein JP, Messingschlager T, Gnambs T, Hutmacher F, Appel M (2024). Attitudes towards AI: measurement and associations with personality. Sci Rep.

[40] Kashdan TB, Disabato DJ, Goodman FR, Doorley JD, McKnight PE (2020). Understanding psychological flexibility: a multimethod exploration of pursuing valued goals despite the presence of distress. Psychol Assess.

[41] Jo D, Im S, Suh DE, Spencer SD, Masuda A (2023). The Personalized Psychological Flexibility Index (PPFI): an item response theory analysis with racially diverse college students. J Psychopathol Behav Assess.

[42] Cherry KM, Hoeven EV, Patterson TS, Lumley MN (2021). Defining and measuring “psychological flexibility”: a narrative scoping review of diverse flexibility and rigidity constructs and perspectives. Clin Psychol Rev.

[43] (2023). A guide to using the accountable health communities health-related social needs screening tool. https://www.cms.gov/priorities/innovation/media/document/ahcm-screeningtool-companion.

[44] Ghaemmaghamfarahani I, Nweke C, Cooper RA (2025). Caregiver identity and resilience across and beyond dementia care: a qualitative study. Innov Aging.

[45] Conde-Sala JL, Reñé-Ramírez R, Turró-Garriga O (2013). Factors associated with the variability in caregiver assessments of the capacities of patients with Alzheimer disease. J Geriatr Psychiatry Neurol.

[46] Fujikawa H, Mori H, Kondo K, Nishizaki Y, Yano Y, Naito T (2026). Adaptation of the Japanese version of the 12-item attitudes towards artificial intelligence scale for medical trainees: multicenter development and validation study. JMIR Med Educ.

[47] Juranek JF, Pochwatko G (2025). Measurement of general attitude toward AI and its relationship with AI anxiety, attitude toward robots and belief in human nature uniqueness: polish adaptation of the ATTARI-12 questionnaire. SSRN.

[48] Han A, Lee HK, Hong I (2025). Relationships among caregiver burden, psychological flexibility processes, and anxiety in family caregivers of people with dementia. Clin Gerontol.

[49] Roberts ET, Mehrotra A (2020). Assessment of disparities in digital access among medicare beneficiaries and implications for telemedicine. JAMA Intern Med.

[50] Gillespie N, Lockey S, Curtis C, Pool J, Akbari A (2023). Trust in artificial intelligence: a global study.

[51] Méndez-Suárez M, Monfort A, Hervas-Oliver JL (2023). Are you adopting artificial intelligence products? Social-demographic factors to explain customer acceptance. Eur Res Manag Bus Econ.

