# Supplementary material for: Psychological and Contextual Factors of AI Acceptance Among Informal Caregivers of People Living With Dementia: Cross-Sectional Pilot Study
Source: JMIR Form Res. 2026 Sep 30;10:e104072. doi: 10.2196/104072 (PMC13628029; doi:10.2196/104072)
Supplement: Supplementary file 1 [file formative-v10-e104072-s001.docx]

Clemson University
Department of Public Health

A STUDY FOR CAREGIVERS

Understanding utilization of caregiver technology

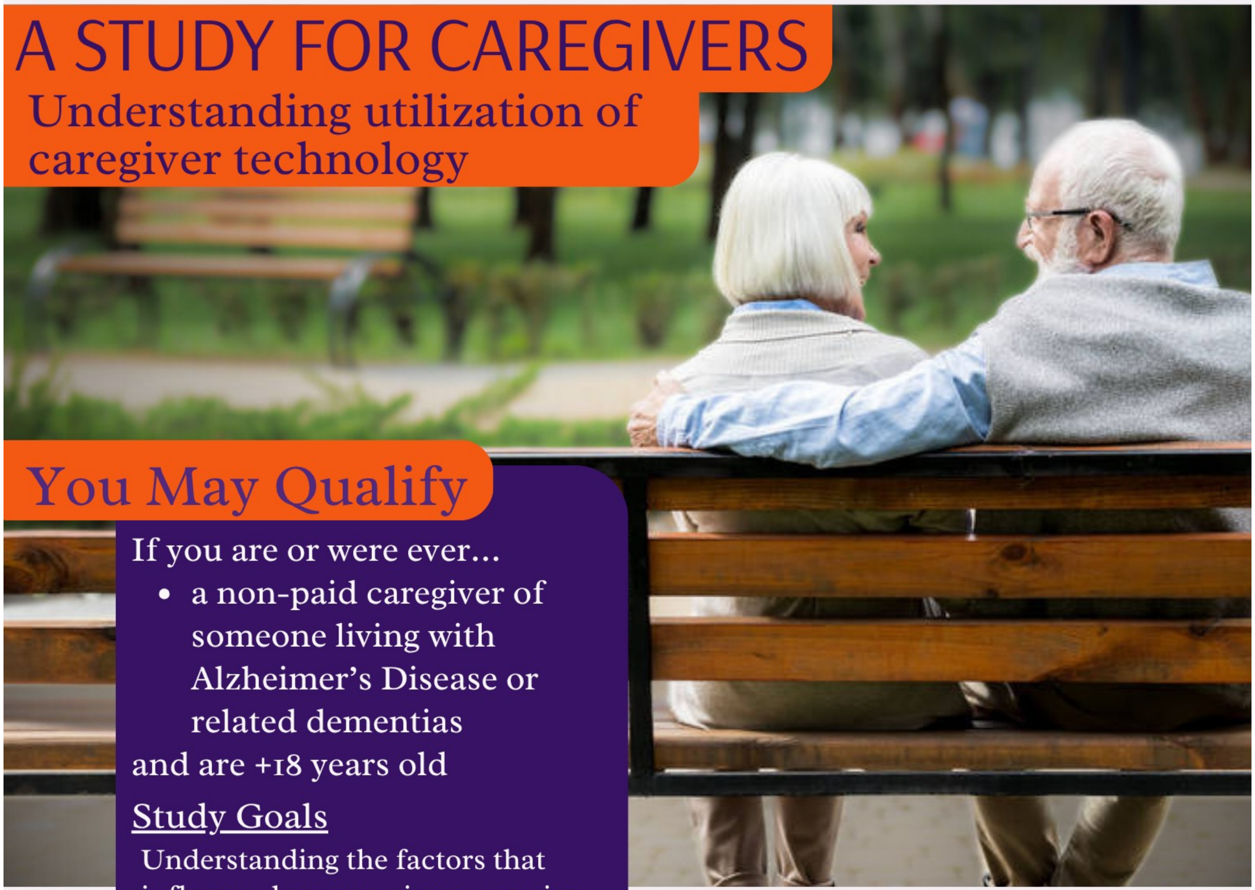

You May Qualify

If you are or were ever...

- a non-paid caregiver of someone living with Alzheimer's Disease or related dementias

and are +18 years old

Study Goals

Understanding the factors that influence how caregivers perceive the use of Artificial Intelligence in applications/technologies aimed at improving their quality of life.

Earn a chance at one of twenty $25 VISA gift cards by participating and completing a short survey!

Take the survey now!

SCAN ME

Study Title

Social and Psychological Factors of AI Acceptance among Informal Caregivers in the United States
PI: Qiping Fan

For more information:
Contact Louis Fisher at
lmfishe@clemson.edu
