# Supplementary material for: Psychological and Contextual Factors of AI Acceptance Among Informal Caregivers of People Living With Dementia: Cross-Sectional Pilot Study
Source: JMIR Form Res. 2026 Sep 30;10:e104072. doi: 10.2196/104072 (PMC13628029; doi:10.2196/104072)
Supplement: Supplementary file 2 [file formative-v10-e104072-s002.docx]

**Table 1. Mean psychological flexibility scores across sociodemographic, caregiver, and social-need subgroups with inter-group comparisons reported as t-test or ANOVA *p*-values.**

|  | | **Psychological Flexibility** | | | | | | | |
| --- | --- | --- | --- | --- | --- | --- | --- | --- | --- |
|  |  | **Total** | ***p*** | **Av** | ***p*** | **Ac** | ***p*** | **H** | ***p*** |
| **Age** | |  |  |  |  |  |  |  |  |
|  | Less than 60 years (n=13) | 64.1 |  | 23.1 |  | 25 |  | 16 |  |
|  | 60+ years (n=18) | 68.2 | 0.332 | 24.5 | 0.601 | 26.6 | 0.331 | 17.2 | 0.546 |
| **Education** | |  |  |  |  |  |  |  |  |
|  | Less than a Bachelor's degree (n=11) | 68.8 |  | 24.7 |  | 26.9 |  | 17.2 |  |
|  | Bachelor's degree  (n=13) | 63.5 |  | 22.8 |  | 24.9 |  | 16.4 |  |
|  | Graduate degree  (n=7) | 68.3 | 0.409 | 24.7 | 0.776 | 26.9 | 0.291 | 16.9 | 0.908 |
| **Perceived illness severity** | | |  |  |  |  |  |  |  |
|  | Severe to very severe (n=9) | 68.8 |  | 23.8 |  | 27.6 |  | 17.4 |  |
|  | Moderately severe (n=13) | 63.7 |  | 24.8 |  | 24 |  | 14.9 |  |
|  | Mild to moderately mild (n=9) | 68.2 | 0.448 | 22.8 | 0.829 | 27 | 0.078 | 18.4 | 0.252 |
| **Marriage status** | |  |  |  |  |  |  |  |  |
|  | Married (n=16) | 67.4 |  | 25.3 |  | 26.4 |  | 15.6 |  |
|  | Separated (n=7) | 65.5 |  | 22.7 |  | 24.6 |  | 18.1 |  |
|  | Never Married (n=8) | 65.4 | 0.874 | 22.1 | 0.551 | 26.1 | 0.624 | 17.3 | 0.564 |
| **Employment status** | |  |  |  |  |  |  |  |  |
|  | Part-time (n=10) | 64.4 |  | 23.7 |  | 25.5 |  | 15.2 |  |
|  | Full-time (n=7) | 70.7 |  | 25.6 |  | 27.1 |  | 18 |  |
|  | Not employed (n=14) | 65.9 | 0.452 | 23.3 | 0.792 | 25.6 | 0.671 | 17.1 | 0.515 |
| **Financial status** | |  |  |  |  |  |  |  |  |
|  | Lower to upper lower (n=8) | 58.6 |  | 22.5 |  | 22.1 |  | 14 |  |
|  | Lower middle (n=13) | 68.9 |  | 23.5 |  | 27.1 |  | 18.3 |  |
|  | Upper middle to upper (n=10) | 69.6 | 0.036 | 25.5 | 0.684 | 27.4 | 0.005 | 16.7 | 0.174 |
| **Housing needs** | |  |  |  |  |  |  |  |  |
|  | Yes (n=9) | 63.9 |  | 21.9 |  | 24.7 |  | 17.3 |  |
|  | No (n=22) | 67.5 | 0.24 | 24.7 | 0.277 | 26.4 | 0.207 | 16.4 | 0.600 |
| **Food insecurity** | |  |  |  |  |  |  |  |  |
|  | Yes (n=7) | 59.4 |  | 19.6 |  | 23.3 |  | 16.6 |  |
|  | No (n=24) | 68.5 | 0.015 | 25.2 | 0.025 | 26.7 | 0.069 | 16.7 | 0.955 |
| **Transport or utility needs** | |  |  |  |  |  |  |  |  |
|  | Yes (n=6) | 62 |  | 21.2 |  | 24.8 |  | 16 |  |
|  | No (n=25) | 67.6 | 0.264 | 24.6 | 0.255 | 26.2 | 0.604 | 16.8 | 0.817 |
