# Supplementary material for: Psychological and Contextual Factors of AI Acceptance Among Informal Caregivers of People Living With Dementia: Cross-Sectional Pilot Study
Source: JMIR Form Res. 2026 Sep 30;10:e104072. doi: 10.2196/104072 (PMC13628029; doi:10.2196/104072)
Supplement: Supplementary file 4 [file formative-v10-e104072-s004.docx]

**Table 1.** Full summary statistics of ANOVA results for AI acceptance (ATTARI-12) across various factors.

| **ATTARI-12** | | **df** | **SS** | **MS** | **F** | ƞ² | ***P*** |
| --- | --- | --- | --- | --- | --- | --- | --- |
|  | Education Level | 2 | 0.876 | 0.4378 | 1.508 | 0.097 | 0.239 |
|  | Error | 28 | 8.126 | 0.2902 |  |  |  |
|  | Perceived Illness severity | 2 | 4.031 | 2.0157 | 11.36 | 0.448 | 0.00025 |
|  | Error | 28 | 4.97 | 0.1775 |  |  |  |
|  | Marriage Status | 2 | 0.608 | 0.304 | 1.014 | 0.068 | 0.376 |
|  | Error | 28 | 8.393 | 0.2998 |  |  |  |
|  | Employment Status | 2 | 0.56 | 0.28 | 0.929 | 0.062 | 0.407 |
|  | Error | 28 | 8.441 | 0.3015 |  |  |  |
|  | Financial Status | 2 | 1.684 | 0.8421 | 3.222 | 0.187 | 0.055 |
|  | Error | 28 | 7.317 | 0.2613 |  |  |  |

**Table 2:** Full summary statistics for ANOVA results for psychological flexibility (PPFI) across various factors.

| **PPFI** | | **df** | **SS** | **MS** | **F** | ƞ² | ***P*** |
| --- | --- | --- | --- | --- | --- | --- | --- |
| **Total** | |  |  |  |  |  |  |
|  | Education Level | 2 | 195.4 | 97.72 | 0.924 | 0.062 | 0.409 |
|  | Error | 28 | 2960.3 | 105.72 |  |  |  |
|  | Perceived Illness severity | 2 | 175.9 | 87.93 | 0.826 | 0.056 | 0.448 |
|  | Error | 28 | 2979.9 | 106.42 |  |  |  |
|  | Marriage Status | 2 | 30.1 | 15.05 | 0.135 | 0.010 | 0.874 |
|  | Error | 28 | 3125.7 | 111.63 |  |  |  |
|  | Employment Status | 2 | 174.2 | 87.1 | 0.818 | 0.055 | 0.452 |
|  | Error | 28 | 2981.5 | 106.5 |  |  |  |
|  | Financial Status | 2 | 668.5 | 334.3 | 3.763 | 0.212 | 0.0357 |
|  | Error | 28 | 2487.2 | 88.8 |  |  |  |
| **Acceptance** | |  |  |  |  |  |  |
|  | Education Level | 2 | 41.7 | 20.86 | 1.289 | 0.084 | 0.291 |
|  | Error | 28 | 453 | 16.18 |  |  |  |
|  | Perceived Illness severity | 2 | 82.5 | 41.24 | 2.801 | 0.167 | 0.0778 |
|  | Error | 28 | 412.2 | 14.72 |  |  |  |
|  | Marriage Status | 2 | 16.4 | 8.185 | 0.479 | 0.033 | 0.624 |
|  | Error | 28 | 478.3 | 17.084 |  |  |  |
|  | Employment Status | 2 | 13.9 | 6.962 | 0.405 | 0.028 | 0.671 |
|  | Error | 28 | 480.8 | 17.171 |  |  |  |
|  | Financial Status | 2 | 154.5 | 77.26 | 6.359 | 0.312 | 0.00529 |
|  | Error | 28 | 340.2 | 12.15 |  |  |  |
| **Avoidance** | |  |  |  |  |  |  |
|  | Education Level | 2 | 28.8 | 14.4 | 0.256 | 0.018 | 0.776 |
|  | Error | 28 | 1575.9 | 56.28 |  |  |  |
|  | Perceived Illness severity | 2 | 21.3 | 10.65 | 0.188 | 0.013 | 0.829 |
|  | Error | 28 | 1583.4 | 56.55 |  |  |  |
|  | Marriage Status | 2 | 67 | 33.48 | 0.61 | 0.041 | 0.551 |
|  | Error | 28 | 1538 | 54.92 |  |  |  |
|  | Employment Status | 2 | 26.6 | 13.27 | 0.235 | 0.017 | 0.792 |
|  | Error | 28 | 1578.2 | 56.36 |  |  |  |
|  | Financial Status | 2 | 43 | 21.49 | 0.385 | 0.027 | 0.684 |
|  | Error | 28 | 1562 | 55.78 |  |  |  |
| **Harnessing** | |  |  |  |  |  |  |
|  | Education Level | 2 | 5.4 | 2.701 | 0.097 | 0.007 | 0.908 |
|  | Error | 28 | 777.4 | 27.763 |  |  |  |
|  | Perceived Illness severity | 2 | 73.4 | 36.7 | 1.449 | 0.094 | 0.252 |
|  | Error | 28 | 709.4 | 25.33 |  |  |  |
|  | Marriage Status | 2 | 31.4 | 15.71 | 0.585 | 0.040 | 0.564 |
|  | Error | 28 | 751.4 | 26.83 |  |  |  |
|  | Employment Status | 2 | 36.2 | 18.12 | 0.68 | 0.046 | 0.515 |
|  | Error | 28 | 746.5 | 26.66 |  |  |  |
|  | Financial Status | 2 | 91.9 | 45.95 | 1.862 | 0.117 | 0.174 |
|  | Error | 28 | 690.9 | 24.67 |  |  |  |

**Table 3:** Full summary statistics of T-test results for AI acceptance (ATTARI-12) across various factors.

| **Variables** | |  | **Diff.** | | **t** | **df** | ***d*** | ***p*** | **Mean Diff. 95% CI** |
| --- | --- | --- | --- | --- | --- | --- | --- | --- | --- |
| Age | |  | 0.14 | | -0.65 | 17.86 | 0.237 | 0.522 | (-0.601, 0.317) |
| Housing needs | |  | 0.12 | | -0.55 | 14.05 | 0.218 | 0.590 | (-0.609, 0.360) |
| Food insecurity | |  | 0.11 | | -0.446 | 9.90 | 0.193 | 0.665 | (-0.446, 0.423) |
| Transport or utility needs | | | 0.12 | | -0.460 | 7.25 | 0.209 | 0.659 | (-0.739, 0.497) |

**Table 4:** Full summary statistics of T-test results for psychological flexibility (PPFI) across various factors.

| **Variables** | |  | **Diff.** | | **t** | **df** | ***d*** | ***p*** | **Mean Diff. 95% CI** |
| --- | --- | --- | --- | --- | --- | --- | --- | --- | --- |
| **Total** | |  |  | |  |  |  |  |  |
|  | Age |  | 4.15 | | 1.00 | 15.79 | 0.364 | 0.332 | (-4.645, 12.935) |
|  | Housing needs | | 3.66 | | 1.20 | 28.48 | 0.475 | 0.240 | (-2.580, 9.893) |
|  | Food Insecurity | | 9.11 | | 2.74 | 14.78 | 1.177 | 0.015 | (2.003, 16.223) |
|  | Transport or utility needs | | 5.56 | | 1.21 | 7.62 | 0.550 | 0.264 | (-5.171, 16.291) |
| **Acceptance** | |  |  | |  |  |  |  |  |
|  | Age |  | 1.56 | | 0.995 | 20.16 | 0.362 | 0.331 | (-1.703, 4.814) |
|  | Housing needs | | 1.74 | | 1.30 | 22.82 | 0.514 | 0.207 | (-1.036, 24.667) |
|  | Food Insecurity | | 3.38 | | 2.04 | 9.80 | 0.876 | 0.069 | (-0.325, 7.087) |
|  | Transport or utility needs | | 1.33 | | 0.55 | 6.05 | 0.250 | 0.605 | (-4.607, 7.260) |
| **Avoidance** | |  |  | |  |  |  |  |  |
|  | Age |  | 1.42 | | 0.529 | 26.11 | 0.193 | 0.602 | (-4.109, 6.955) |
|  | Housing needs | | 2.84 | | 1.12 | 20.28 | 0.443 | 0.277 | (-2.727, 8.139) |
|  | Food Insecurity | | 5.60 | | 2.46 | 17.30 | 1.057 | 0.025 | (0.795, 10.295) |
|  | Transport or utility needs | | 3.39 | | 1.21 | 9.66 | 0.550 | 0.255 | (-2.886, 9.672) |
| **Harnessing** | |  |  | |  |  |  |  |  |
|  | Age |  | 1.17 | | 0.61 | 24.45 | 0.222 | 0.547 | (-2.769, 5.102) |
|  | Housing needs | | 0.92 | | -0.53 | 22.15 | 0.210 | 0.600 | (-4.526, 2.678) |
|  | Food Insecurity | | 0.14 | | 0.06 | 9.06 | 0.026 | 0.955 | (-5.207, 5.481) |
|  | Transport or utility needs | | 0.84 | | 0.242 | 5.66 | 0.110 | 0.817 | (-7.784, 9.464) |
