# Supplementary material for: Psychological and Contextual Factors of AI Acceptance Among Informal Caregivers of People Living With Dementia: Cross-Sectional Pilot Study
Source: JMIR Form Res. 2026 Sep 30;10:e104072. doi: 10.2196/104072 (PMC13628029; doi:10.2196/104072)
Supplement: Supplementary file 5 [file formative-v10-e104072-s005.docx]

**Table 1.** Sensitivity analysis of ANOVAs between the full sample (past and current caregivers) vs current caregivers only.

|  |  | **Full Sample (n=31)** | **Current Caregivers (n=21)** |
| --- | --- | --- | --- |
|  | | ***p*-value** | ***p*-value** |
| Illness Severity | | <0.001 | 0.001 |
| Financial Status | | 0.055 | 0.0358 |

**Table 2.** Sensitivity analysis of linear regressions between the full sample (past and current caregivers) vs current caregivers only.

|  | | **Full Sample (n=31)** | **Current Caregivers (n=21)** |
| --- | --- | --- | --- |
|  | | **β (95% CI) *p*** | **β (95% CI) *p*** |
| **Psychological Flexibility** | |  |  |
|  | PPFIt | 0.015 (-0.004, 0.035) *0.123* | 0.02 (-0.002, 0.042) *0.08* |
|  | PPFIac | 0.050 (0.002, 0.097) *0.042* | 0.050 (-0.007, 0.112) *0.08* |
|  | PPFIav | -0.002 (-0.031, 0.026) *0.866* | 0.003 (-0.032, 0.040) *0.832* |
|  | PPFIh | 0.034 (-0.004, 0.073) *0.079* | 0.038 (-0.003, 0.080) *0.067* |
| **Illness Severity (Mild to moderately mild)** | | |  |
|  | Moderately severe | -0.415 (-0.789, -0.040) *0.031* | -0.404 (-0.779, -0.029) *0.036* |
|  | Severe to very severe | 0.454 (0.047, 0.861) *0.030* | 0.701 (0.167, 1.236) *0.013* |
| **Financial Status (Upper middle to upper)** | | |  |
|  | Lower to upper lower | -0.467 (-0.963, 0.030) *0.065* | -0.242 (-0.791, 0.307) *0.367* |
|  | Lower middle | 0.101 (-0.340, 0.541) *0.643* | 0.404 (-0.145, 0.953) *0.139* |
