# Supplementary material for: Psychological and Contextual Factors of AI Acceptance Among Informal Caregivers of People Living With Dementia: Cross-Sectional Pilot Study
Source: JMIR Form Res. 2026 Sep 30;10:e104072. doi: 10.2196/104072 (PMC13628029; doi:10.2196/104072)
Supplement: Supplementary file 7 [file formative-v10-e104072-s007.docx]

**STROBE Statement—checklist of items that should be included in reports of observational studies**

|  | **Item No.** | **Recommendation** | **Page  No.** | **Relevant text from manuscript** |
| --- | --- | --- | --- | --- |
| **Title and abstract** | 1 | (*a*) Indicate the study’s design with a commonly used term in the title or the abstract | 1 | Social and Psychological Factors of AI Acceptance Among Informal Caregivers of People Living with Dementia: A Cross-Sectional Survey |
|  |  | (*b*) Provide in the abstract an informative and balanced summary of what was done and what was found | 1 | A cross-sectional web-based survey was administered via Qualtrics…. |
| **Introduction** | | | |  |
| Background/rationale | 2 | Explain the scientific background and rationale for the investigation being reported | 3-5 | Considering the theoretical framework of interconnections…more research needs to be done on measuring levels of PF and its correlation to AI acceptance in ICs. |
| Objectives | 3 | State specific objectives, including any prespecified hypotheses | 6 | Accordingly, this study seeks…. |
| **Methods** | | | |  |
| Study design | 4 | Present key elements of study design early in the paper | 6 | This was a cross-sectional, web-based survey study… |
| Setting | 5 | Describe the setting, locations, and relevant dates, including periods of recruitment, exposure, follow-up, and data collection | 6 | The survey was open from mid-March to mid-April, 2025… |
| Participants | 6 | (*a*) *Cohort study*—Give the eligibility criteria, and the sources and methods of selection of participants. Describe methods of follow-up  *Case-control study*—Give the eligibility criteria, and the sources and methods of case ascertainment and control selection. Give the rationale for the choice of cases and controls  *Cross-sectional study*—Give the eligibility criteria, and the sources and methods of selection of participants | 7 | Participants were recruited using electronic flyers…  The inclusion criteria for caregivers in this study were that… |
|  |  | (*b*) *Cohort study*—For matched studies, give matching criteria and number of exposed and unexposed  *Case-control study*—For matched studies, give matching criteria and the number of controls per case | N/a |  |
| Variables | 7 | Clearly define all outcomes, exposures, predictors, potential confounders, and effect modifiers. Give diagnostic criteria, if applicable | 6 | Participants were sent a Qualtrics survey to screen leads for eligibility, assess their attitude towards AI acceptance, and evaluate various factors that may influence their perception. |
| Data sources/ measurement | 8* | For each variable of interest, give sources of data and details of methods of assessment (measurement). Describe comparability of assessment methods if there is more than one group | 7 | Study Messures… |
| Bias | 9 | Describe any efforts to address potential sources of bias | 7 | The survey contained questions that automatically screened |
| Study size | 10 | Explain how the study size was arrived at | 9 | A total of 31 informal caregivers completed all survey items |

Continued on next page

| Quantitative variables | 11 | Explain how quantitative variables were handled in the analyses. If applicable, describe which groupings were chosen and why | 9 | Analyses were conducted in… |
| --- | --- | --- | --- | --- |
| Statistical methods | 12 | (*a*) Describe all statistical methods, including those used to control for confounding | 9 | Analyses were conducted in… |
|  |  | (*b*) Describe any methods used to examine subgroups and interactions | 9 | Bivariate comparisons… |
|  |  | (*c*) Explain how missing data were addressed | 9 | …completed all survey items… |
|  |  | (*d*) *Cohort study*—If applicable, explain how loss to follow-up was addressed  *Case-control study*—If applicable, explain how matching of cases and controls was addressed  *Cross-sectional study*—If applicable, describe analytical methods taking account of sampling strategy | N/a |  |
|  |  | (*e*) Describe any sensitivity analyses |  |  |
| **Results** | | | | |
| Participants | 13* | (a) Report numbers of individuals at each stage of study—eg numbers potentially eligible, examined for eligibility, confirmed eligible, included in the study, completing follow-up, and analysed | 8 | As seen in Figure 2, out of 876 research leads that were emailed for study recruitment… |
|  |  | (b) Give reasons for non-participation at each stage | 8 |  |
|  |  | (c) Consider use of a flow diagram | 8 |  |
| Descriptive data | 14* | (a) Give characteristics of study participants (eg demographic, clinical, social) and information on exposures and potential confounders | 10 | Table 1: Caregiver demographics and characteristic |
|  |  | (b) Indicate number of participants with missing data for each variable of interest | 9 | A total of 31 informal caregivers completed all survey items |
|  |  | (c) *Cohort study*—Summarise follow-up time (eg, average and total amount) | N/a |  |
| Outcome data | 15* | *Cohort study*—Report numbers of outcome events or summary measures over time | N/a |  |
|  |  | *Case-control study—*Report numbers in each exposure category, or summary measures of exposure | N/a |  |
|  |  | *Cross-sectional study—*Report numbers of outcome events or summary measures | 12 | Table 3 |
| Main results | 16 | (*a*) Give unadjusted estimates and, if applicable, confounder-adjusted estimates and their precision (eg, 95% confidence interval). Make clear which confounders were adjusted for and why they were included | 15 | Tables 4 and 5 |
|  |  | (*b*) Report category boundaries when continuous variables were categorized | 9 and 15 | ATTARI-12 scores greater than 3.0 were considered acceptant, while 3.0 or lower were not acceptant of AI |
|  |  | (*c*) If relevant, consider translating estimates of relative risk into absolute risk for a meaningful time period | N/a |  |

Continued on next page

| Other analyses | 17 | Report other analyses done—eg analyses of subgroups and interactions, and sensitivity analyses | 16 | Sensitivity analyses through the restriction of all primary analyses |
| --- | --- | --- | --- | --- |
| **Discussion** | | | | |
| Key results | 18 | Summarise key results with reference to study objectives | 17 | moderate ATTARI-12 score (3.01/5) demonstrated a neutral attitude regarding AI use  AI acceptance varied most with caregiver-rated dementia severity and perceived financial status |
| Limitations | 19 | Discuss limitations of the study, taking into account sources of potential bias or imprecision. Discuss both direction and magnitude of any potential bias | 19 | This study had several limitations… |
| Interpretation | 20 | Give a cautious overall interpretation of results considering objectives, limitations, multiplicity of analyses, results from similar studies, and other relevant evidence | 20 | …our study demonstrates that caregivers’ acceptance of such technology may be influenced by their financial status and their care recipient’s disease severity. |
| Generalisability | 21 | Discuss the generalisability (external validity) of the study results | 19 | Firstly, the smaller sample size (n=31) limits generalizability and precludes causal inference. |
| **Other information** | |  | | |
| Funding | 22 | Give the source of funding and the role of the funders for the present study and, if applicable, for the original study on which the present article is based | 21 | This study was supported and funded by… |

*Give information separately for cases and controls in case-control studies and, if applicable, for exposed and unexposed groups in cohort and cross-sectional studies.

**Note:** An Explanation and Elaboration article discusses each checklist item and gives methodological background and published examples of transparent reporting. The STROBE checklist is best used in conjunction with this article (freely available on the Web sites of PLoS Medicine at http://www.plosmedicine.org/, Annals of Internal Medicine at http://www.annals.org/, and Epidemiology at http://www.epidem.com/). Information on the STROBE Initiative is available at www.strobe-statement.org.
